# Driving Revisit Intentions Through Medical Information and Service Quality in General Hospitals: An Extended Technology Acceptance Model Approach

**DOI:** 10.3390/healthcare14142045

**Published:** 2026-07-08

**Authors:** Jebum Kim, SangYoon Lim

**Affiliations:** 1 Department of Global Culture and Management, Calvin University, Yongin-si 16911, Republic of Korea; jbkim@calvin.ac.kr; 2 Healthcare Administration Team, Seoul National University Bundang Hospital, Seongnam-si 13620, Republic of Korea

**Keywords:** medical information quality, medical service quality, technology acceptance model (TAM), patient satisfaction, revisit intention

## Abstract

Background: As Information and Communication Technology (ICT) advances, the healthcare market is shifting toward a consumer-centered paradigm. This study analyzes the structural relationships between medical information quality, medical service quality, and Technology Acceptance Model (TAM) variables to determine their impact on patient satisfaction and revisit intentions in a general hospital context. Methods: An online survey was conducted with 376 consumers who had experience using general hospitals in South Korea between June and December 2024. Data were analyzed using structural equation modeling (SEM) with SPSS 29.0 and AMOS 29.0. Ethical measures, including informed consent and anonymity, were strictly followed. Results: Findings indicate that among medical information quality factors, accuracy significantly enhanced perceived usefulness, while timeliness positively influenced perceived ease of use. Regarding medical service quality, both accessibility and responsiveness significantly improved both usefulness and ease of use, with responsiveness being the most powerful predictor of ease of use (β = 0.655). While both TAM variables significantly increased patient satisfaction, only perceived ease of use and satisfaction directly drove revisit intentions; perceived usefulness influenced revisit intention only through the mediation of satisfaction. Conclusions: Patient satisfaction is a paramount factor directly influencing loyalty. Healthcare administrators should prioritize the accuracy and timeliness of digital health information while improving service responsiveness to enhance long-term hospital competitiveness in the proactive consumer era.

## 1. Introduction

With the remarkable advancement of Information and Communication Technologies (ICT), internet usage among consumers has increased dramatically. According to the 2022 Survey on the Internet Usage by the National Information Society Agency, it was analyzed that approximately 93% of South Korean citizens use the internet. Furthermore, an analysis of internet usage rates by age group over the past five years showed that the usage rate for those in their 30s to 60s all exceeded 90%, while for those aged 70 and over, it increased from 38.6% in 2018 to 54.7% in 2022, indicating a rise in internet usage among the elderly as well [1].

As consumers’ internet usage increases, their level of information exploration has risen daily, enabling them to search, compare, and analyze various types of data they desire online independently. Moreover, medical consumers can now decide for themselves what treatment to seek based on the information acquired. Simultaneously, as social and economic standards have improved, interest in and demand for health have increased, and individuals are dedicated to maintaining and promoting health for a quality life. Today, consumers seeking medical services at institutions collect and analyze desired medical information, select medical institutions using information beneficial to them, and further share this information with close acquaintances or family [2].

Regarding the above, prior research indicates that 70.5% of internet users search for health and medical information. By age group, the 30s recorded the highest figure at 80.6%, followed by 80.2% for the 40s, 78.3% for the 50s, 76.1% for the 60s, and 72.4% for the 20s [1]. As consumers’ activities in collecting and analyzing medical information through exploration increase, many changes have occurred in the healthcare system. Previously, consumers had no choice but to collect and utilize limited information provided by a supplier-centered medical market and take a passive attitude. However, the development of information technology has shifted the medical market to be consumer-centered, and consequently, consumers have adopted a proactive attitude [3]. In other words, before using services, many medical consumers search, collect, and analyze medical information to select and purchase medical institutions that fit their needs.

As the medical field shifts toward a consumer-centered system, medical consumers have begun to demand high-quality medical services and evaluate the quality of services provided by institutions [3]. The core of this change is that necessary medical information can now be collected without time or place constraints amidst vast amounts of information, and there is an increase in consumers demanding high-quality services to use rising medical costs efficiently [3]. Furthermore, for patients requiring high-cost treatments or examinations, the need for medical institutions to provide quality information and services to these consumers is increasing as they demand or require high-quality information and services. Accordingly, this study aims to conduct research on medical information quality and medical service quality targeting consumers with experience using medical institutions. Specifically, focusing on consumers with experience in general hospitals, this study analyzes how useful and easy the relevant medical information and services are to suggest the necessity of providing helpful information and services to consumers in need.

While the Technology Acceptance Model (TAM) was originally developed to explain the adoption of specific software or hardware systems, its application to the healthcare service environment is highly justified in the digital era. Modern medical consumers do not merely receive treatment; they actively interact with a hospital’s digital ecosystem—including online reservation systems, mobile healthcare applications, medical information portals, and automated administrative channels—before and during their physical visit. Therefore, navigating general hospital information and services represents a technology-mediated consumer experience. To clarify the mechanism of this acceptance, this study establishes the following research questions (RQs):

RQ1: How do medical information quality (timeliness and accuracy) and medical service quality (accessibility and responsiveness) shape consumers’ internal beliefs (perceived usefulness and perceived ease of use) within a general hospital’s service delivery ecosystem?

RQ2: How do these internal TAM beliefs subsequently drive patient satisfaction and long-term revisit intentions?

## 2. Theoretical Background

### 2.1. Medical Information Quality

Medical information refers to comprehensive data including information on treatments and examinations provided by institutions, customer details such as name, address, and gender, and institutional information like facilities, departments, staff, and location. Medical information quality is defined as the degree to which medical consumers perceive the value of the information acquired through exploration. High-quality medical information is characterized by treatment cost reduction, prevention of medical accidents, and superior treatment quality [4]. With the recent development of IT and the increase in internet usage, consumers can now collect medical information without time or location constraints, and many have begun to collect and analyze information necessary for themselves [5]. This is because consumers’ information utilization needs have diversified, such as the desire for high-quality information, selecting the best institution, rational medical spending, and acquiring knowledge, especially for those needing treatment where health and life are directly related.

Regarding this, prior research found that over 80% of adults in the US search for medical information via the internet and 70.5% in South Korea do so [1]. As the number of consumers exploring and utilizing medical information increases, the importance of its quality is emphasized. In particular, while high-quality medical information can promote health, it can also cause life-threatening problems, making the importance of quality even higher [6]. In this regard, the WHO [7] published guidelines for healthcare information quality, stating that factors such as accuracy, reliability, importance, validity, readability, completeness, timeliness, confidentiality, and accessibility must be met. Recent studies specifically analyzing latest papers have identified timeliness and accuracy as critical elements [8,9]. Therefore, this study refers to prior research to categorize medical information quality into timeliness and accuracy to analyze perceived usefulness and ease of use.

### 2.2. Medical Service Quality

Medical service refers to services provided by institutions and professionals, which consumers judge based on their experience. Medical service quality means that consumers’ expectations are met, leading to trust and satisfaction, and it is evaluated as a crucial factor influencing revisit intention. That is, it is a comprehensive evaluation where expectations are met and satisfaction is formed [10]. This quality differs from general service quality; while general quality may be decided at the moment of experience, medical quality is often determined by treatment results after a certain period following the service, and is characterized by being determined by the provider [11].

Service quality is a key factor in choosing an institution. Since it significantly affects satisfaction, loyalty, and ultimately profit, measuring and improving it is of utmost importance [12]. A typical model for measurement is SERVQUAL, consisting of 5 factors (tangibility, reliability, responsiveness, empathy, and assurance) and 22 items [13]. Many studies use SERVQUAL to measure medical service quality. However, because the concept in the health sector ranges from broad health promotion to narrow treatment, definitions and components vary by researcher. Recent studies emphasize that accessibility and responsiveness are important factors for consumers [14,15]. Thus, this study categorizes medical service quality into accessibility and responsiveness.

Although the classic SERVQUAL model encompasses five distinct dimensions, this study selectively focuses on accessibility and responsiveness to ensure model parsimony and context-specific relevance. In high-volume, complex healthcare settings like South Korean general hospitals, the primary administrative friction points for patients revolve around physical/digital entry and communication speed. Accessibility captures the ease of navigating the hospital’s physical location and digital infrastructure, while responsiveness evaluates the promptness of staff communication and administrative procedures. Other dimensions, such as empathy or tangibles, were intentionally excluded as they overcomplicate the structural model without significantly contributing to the immediate cognitive appraisal of technology-mediated service acceptance.

### 2.3. Technology Acceptance Model (TAM)

The Technology Acceptance Model (TAM) was first established by Davis in 1989 to effectively explain the process of accepting or adopting specific technologies and systems [16]. It uses internal belief variables, perceived usefulness and perceived ease of use, to analyze influences from external variables, significantly affecting attitudes and behavioral intentions [16]. Perceived usefulness means that using a system helps achieve a user’s goal with good results, while perceived ease of use means being able to use it with minimal effort [16,17]. These variables are widely studied in healthcare [18,19]. As technologies evolved, TAM also changed, excluding variables with weak mediation like attitude or adding various external variables [17]. Recently, due to COVID-19, the need for reliable, useful, and easy medical information and services has increased. Thus, this study utilizes TAM to analyze perceived usefulness and ease.

### 2.4. Satisfaction

Satisfaction is defined as consumers feeling positive emotions when their expectations are met, and in a medical context, it is when expectations regarding information and services are fulfilled [20]. Since its purpose is health and treatment, it differs from general satisfaction and is highly related to quality. Consumers may evaluate satisfaction differently based on treatment results, and institutions use it as a key indicator for maintaining relationships. In short, satisfaction is formed according to the quality experienced [21]. Prior research shows that high quality has a positive effect on satisfaction, leading to emotional bonds and trust for long-term relationships [22,23]. Maintaining existing patients is more profitable than finding new ones, thus highlighting the need to provide satisfaction.

### 2.5. Revisit Intention

Revisit intention refers to planned future behavior where beliefs and attitudes lead to actual purchases. In the medical field, it is a patient’s direct evaluation of whether they will visit the institution again, used as a management indicator [24]. It is the final evaluation where satisfied patients usually return. Prior research indicates that satisfied patients trust the institution and visit continuously for treatment [25], and providing accurate information is an effective strategy for long-term relationships [26]. Revisit intention is crucial for revenue and is a key performance goal. Since retaining consumers requires less effort than attracting new ones, it is a vital element for management.

## 3. Research Methodology

### 3.1. Research Model

This study aims to research medical information quality and service quality targeting consumers with experience in general hospitals. It analyzes how useful and easy these are to suggest the necessity of providing helpful information and services.

### 3.2. Research Hypotheses

#### 3.2.1. Relationship Between Medical Information Quality and Perceived Usefulness/Ease of Use

Medical information quality refers to perceiving value in information acquired through exploration; high quality is characterized by cost reduction and accident prevention [4]. With IT development, consumers now collect information necessary for themselves [5]. Its importance is emphasized as information can promote health or cause fatal problems [6]. Thus, consumers search for useful and easy information [27]. Prior research analyzed that information quality significantly affects usefulness and ease [28], especially timeliness and accuracy [8,9].

**H1.**  *Medical information quality significantly and positively (+) affects perceived usefulness. (H1a: Timeliness, H1b: Accuracy).*

**H2.**  *Medical information quality significantly and positively (+) affects perceived ease of use. (H2a: Timeliness, H2b: Accuracy).*

#### 3.2.2. Relationship Between Medical Service Quality and Perceived Usefulness/Ease of Use

Medical service quality refers to the degree to which expectations are met and satisfaction is formed [10]. Unlike general services, it is often determined by results after a period of time and the provider [11]. Consumers utilize useful and easy service quality, and institutions strive for satisfaction. Prior research shows service quality affects usefulness and ease [28], with accessibility and responsiveness being important [14,15].

**H3.**  *Medical service quality significantly and positively (+) affects perceived usefulness. (H3a: Accessibility, H3b: Responsiveness).*

**H4.**  *Medical service quality significantly and positively (+) affects perceived ease of use. (H4a: Accessibility, H4b: Responsiveness).*

#### 3.2.3. Relationship Between PU/PEOU and Satisfaction/Revisit Intention

TAM uses internal beliefs (usefulness, ease) to analyze external influences, affecting attitudes and intentions [16]. Usefulness means achieving goals, while ease means using it with minimal effort [16,17]. These are widely studied in healthcare [18,19]. Research on E-health services during COVID-19 and telemedicine apps found usefulness and ease positively affect satisfaction [29,30]. Other studies analyzed the relationship between PU/PEOU and revisit intention [31]. Recently, the need for useful and easy information and services has been emphasized.

**H5.**  *Perceived usefulness/ease significantly and positively (+) affects satisfaction. (H5a: PU, H5b: PEOU).*

**H6.**  *Perceived usefulness/ease significantly and positively (+) affects revisit intention. (H6a: PU, H6b: PEOU).*

#### 3.2.4. Relationship Between Satisfaction and Revisit Intention

Satisfaction in a medical context is when expectations for information and services are met [20]. Consumers evaluate satisfaction based on results, and institutions use it to maintain relationships, because satisfied consumers return. Prior research indicates satisfaction significantly affects revisit intention [26], including in public hospitals [24].

**H7.**  *Satisfaction significantly and positively (+) affects revisit intention.*

**Figure 1:**
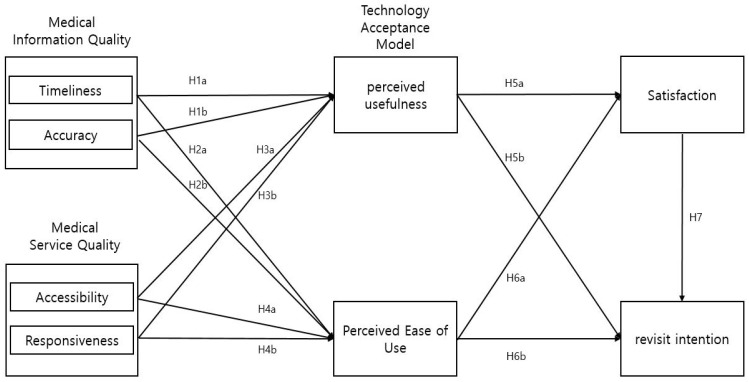
Research Model.

The proposed research model illustrating these hypothesized relationships is presented in Figure 1.

### 3.3. Operational Definition of Variables

This study targets general hospital users. Definitions are in Table 1. A total of 32 items were set based on prior research, using a 5-point Likert scale. Timeliness was measured with 4 items (up-to-date, timely, periodic updates), and accuracy with 4 items (correct, clear, error-free). Service accessibility used 4 items (location, transport, parking) and responsiveness used 4 items (easy explanations, quick staff answers, fast procedures). Usefulness used 4 items (helpful, beneficial, solving queries) and ease used 4 items (simple, proficient use, low effort). Satisfaction used 4 items (satisfaction with prescription, staff, value) and revisit intention used 4 items (continuous use, priority, use even if far).

### 3.4. Data Collection and Analysis

The empirical data for this study were gathered through a structured online survey administered by a specialized commercial research panel provider in South Korea from 1 June to 18 December 2024. To ensure demographic representativeness and quality control, a non-probability quota sampling method based on age and gender was utilized. Strict respondent inclusion criteria were enforced: participants were required to be adults (aged 20 or older) who had at least one direct utilizing experience with a general hospital in South Korea as an outpatient (*n* = 214, 56.9%) or an inpatient (*n* = 162, 43.1%) within the past 12 months prior to the survey date. Respondents who did not meet these temporal or experiential criteria were automatically screened out. Regarding the adequacy of the sample size, the final valid sample of 376 respondents is highly justified for structural equation modeling (SEM) analysis. According to established methodological criteria in multivariate analysis, a minimum sample size of 200, or a ratio of 10 to 20 cases per estimated parameter, is widely accepted as sufficient for stable maximum likelihood estimation. Given that our structural model evaluates 8 latent constructs with 32 observed variables, a sample size of *N* = 376 comfortably satisfies these rigorous parameters, ensuring adequate statistical power and stable standard errors during the SEM procedures executed via SPSS 29.0 and AMOS 29.0 (IBM Corp., Armonk, NY, USA).

### 3.5. Ethical Considerations

This study was conducted in accordance with the ethical principles of the Declaration of Helsinki. Since this research involved an anonymous online survey posing minimal risk to subjects and collected no sensitive personal or personally identifiable information (PII), it qualified for an institutional review exemption under Article 15, Paragraph 2 of the Bioethics and Safety Act of the Republic of Korea and Article 13 of its Enforcement Rule. Under these national statutory criteria, non-interventional social-behavioral studies with completely anonymized datasets are categorically exempt from formal Institutional Review Board (IRB) review. Consequently, no specific institutional approval code or formal written waiver document was issued by an ethics committee, and written documentation of the exemption determination is unavailable. Statistical data collection was initiated only after obtaining electronic informed consent from each of the 376 participants, and complete respondent anonymity was strictly maintained throughout the study.

## 4. Analysis Results

### 4.1. Demographic Characteristics

Demographics are in Table 2: 206 males (54.8%), 170 females (45.2%). Users in their 30s were most common at 156 (41.5%), then 20s (19.4%), 40s (17.3%), 50s (12.8%), and 60s+ (9.0%). A total of 199 (52.9%) were office workers; 155 (41.2%) earned 3–4 million KRW.

### 4.2. Reliability and Validity

Results are in Table 3. Cronbach’s α values were all >0.7, ensuring reliability. Convergence validity was secured with AVE > 0.5 and CR > 0.7. Model fit (χ^2^/df = 2.883, GFI = 0.887, TLI = 0.930, CFI = 0.938, SRMR = 0.027, RMSEA = 0.074) met standards. Discriminant validity was confirmed as AVE square roots were higher than correlation coefficients (Table 4).

To evaluate the potential threat of common method bias (CMB) inherent in this self-reported, cross-sectional survey design, Harman’s single-factor test was performed. The unrotated factor analysis revealed that a single dominant factor accounted for 61.71% of the total variance. Because this value exceeds the conventional 50% threshold, it serves as a potential warning sign indicating that common method variance may be present in the dataset. However, while this statistical threshold demands cautious interpretation, the subsequent confirmatory factor analysis (CFA) and the rigorous discriminant validity assessment (Table 4), wherein the square roots of the AVE for all constructs are substantially higher than their inter-construct correlations, demonstrate that the latent constructs remain empirically distinct and identifiable within the structural framework. While the Fornell–Larcker criteria do not completely eliminate the possibility of bias, they indicate that the structural relationships are driven by genuine construct variances rather than being purely artificial artifacts of the measurement method.

### 4.3. Hypothesis Testing Results

All path estimates reported in this study are standardized coefficients (β). The structural model demonstrated substantial explanatory power for the endogenous variables; the predictors accounted for 67.2% of the variance in Perceived Usefulness (R^2^ = 0.672), 74.3% in Perceived Ease of Use (R^2^ = 0.743), 69.6% in Patient Satisfaction (R^2^ = 0.696), and 51.0% in Revisit Intention (R^2^ = 0.510). The results of the structural equation modeling (SEM) analysis used to verify the hypothesized relationships are summarized in Table 5. Additionally, the statistical estimates for the indirect and mediation effects analyzed via bootstrapping are presented in Table 6.

The results of the hypothesis testing are as follows:

Regarding medical information quality, Accuracy (H1b: β = 0.338, *p* < 0.001) significantly influenced perceived usefulness, whereas Timeliness (H1a) did not show a statistically significant effect. Conversely, for perceived ease of use, Timeliness (H2a: β = 0.206, *p* < 0.001) was a significant predictor, while Accuracy (H2b) had no significant impact.

In terms of medical service quality, both Accessibility (H3a: β = 0.261, *p* < 0.001) and Responsiveness (H3b: β = 0.258, *p* < 0.001) were found to have significant positive effects on perceived usefulness. Similarly, both Accessibility (H4a: β = 0.135, *p* = 0.003) and Responsiveness (H4b: β = 0.655, *p* < 0.001) significantly influenced perceived ease of use. Notably, responsiveness emerged as the most powerful determinant of perceived ease of use.

Furthermore, both internal belief variables of the Technology Acceptance Model Perceived Usefulness (H5a: β = 0.652, *p* < 0.001) and Perceived Ease of Use (H5b: β = 0.229, *p* < 0.001) significantly increased patient satisfaction. While Perceived Ease of Use (H6b: β = 0.261, *p* < 0.001) directly influenced revisit intention, Perceived Usefulness (H6a) did not have a direct effect. Finally, Satisfaction (H7: β = 0.527, *p* < 0.001) was confirmed to be a critical antecedent that significantly drives revisit intention.

## 5. Discussion

### 5.1. Research Results

This study aimed to investigate the impact of medical information quality and medical service quality on proactive consumers with experience in general hospitals. The empirical findings of this study are as follows:

First, regarding medical information quality, the structural equation model revealed a nuanced, non-reciprocal relationship between quality dimensions and TAM belief variables. Specifically, Accuracy significantly influenced Perceived Usefulness (PU), whereas Timeliness failed to demonstrate a statistically significant direct impact on PU. Conversely, for Perceived Ease of Use (PEOU), Timeliness emerged as a significant predictor, while Accuracy did not exhibit a statistically significant effect. These non-significant findings counter the overgeneralized logic of prior assumptions and offer critical insights into modern healthcare consumer behavior. The non-significant path from timeliness to usefulness indicates that in a mature digital environment, the real-time or prompt update of online medical data is perceived by consumers as a baseline hygiene factor (a mandatory prerequisite) rather than an added value that enhances the core utility of the information; however, as supported by the significant path to PEOU, when information is updated in a timely manner, it reduces the cognitive effort required to search for updates, aligning with digital health communication trends [32,33]. Similarly, the non-significant path from accuracy to PEOU demonstrates that while the correctness of medical data is paramount for its cognitive evaluation as beneficial and useful (driving PU) especially given that unverified online health information often suffers from quality deficiencies and inaccuracies [34,35], the absolute veracity of the data does not structurally alter or ease the technical interface navigation or the physical process of information processing (PEOU) for the user. These empirical insights suggest that healthcare administrators must carefully segment information quality dimensions rather than treating them as a monolithic construct.

Second, regarding medical service quality, both Accessibility and Responsiveness were confirmed to have significant positive effects on both PU and PEOU. Specifically, accessibility was a key driver of PU, while responsiveness emerged as the most powerful predictor of PEOU (β = 0.655, *p* < 0.001). This indicates that consumers perceive healthcare services as useful when they are accessible and find them easy to use when the staff responds promptly. This is consistent with prior research showing that responsiveness significantly increases perceived usefulness [36] and that accessibility is fundamentally linked to the perceived utility of healthcare services [37].

Third, both PU and PEOU were found to have significant positive effects on Satisfaction. This implies that hospital consumers feel satisfied when they perceive the medical information and services provided to be both useful and easy to navigate. These findings are supported by existing literature which demonstrates that both internal belief variables of the Technology Acceptance Model are statistically significant antecedents of user satisfaction [38,39].

Fourth, while PEOU had a significant positive effect on Revisit Intention, PU did not show a direct significant impact. This important finding reveals that the sheer clinical utility or usefulness of a hospital’s information and services is an expected minimum standard that does not automatically translate into institutional loyalty. Instead, long-term retention and future behavioral intent are heavily driven by the convenience and frictionless nature of the service experience (PEOU). More importantly, the non-significant direct path from PU to revisit intention, coupled with the highly significant path from PU to satisfaction, underscores a full mediation mechanism. This statistically demonstrates that highly useful medical information and service systems must first be synthesized into emotional Patient Satisfaction before they can effectively trigger a consumer’s behavioral commitment to return to the healthcare institution. This empirical insight underscores the vital role of patient satisfaction as a primary conduit for institutional loyalty [39,40].

Fifth, Satisfaction was confirmed to have a significant positive effect on Revisit Intention. Satisfied medical consumers develop trust in the institution and continue to visit for treatment. This is consistent with evidence that institutional trust built through satisfaction leads to persistent hospital visits [25] and that satisfaction is one of the most vital factors influencing the intention to return to a medical facility [24].

### 5.2. Implications

The academic and practical implications derived from the results of this study are as follows:

First, this study suggests the necessity of developing strategies that can satisfy the timeliness and accuracy of medical information for consumers seeking to utilize healthcare institutions, particularly general hospitals. Our analysis revealed that the timeliness of medical information quality did not significantly influence perceived usefulness, and accuracy did not significantly influence perceived ease of use. This indicates that consumers who intend to use general hospitals do not perceive the medical information they have searched for as being utilized in a timely manner or as being inherently accurate.

These results are influenced not only by individual medical consumers but also by broader social factors. In South Korea, with the advancement of information and communication technology and increasing internet usage, it has been analyzed that more than 90% of the population utilizes the internet [1]. Consequently, an environment has been established where consumers can search for and analyze the data they desire; specifically, medical consumers seeking healthcare institutions can now select providers based on the information they have acquired. Furthermore, as environments for utilizing online artificial intelligence (AI) chatbots such as ChatGPT (14 March 2023, version; OpenAI, San Francisco, CA, USA) have been created, and platforms like YouTube, Bilibili, and TikTok enable information search and knowledge acquisition, a society has emerged where information can be accessed and utilized without temporal or spatial restrictions [41,42]. However, at the same time, as consumers are exposed to vast amounts of information, issues regarding the quality of medical information have begun to emerge [43]. Problems such as wasting time analyzing whether the acquired information is helpful, thereby failing to utilize information in a timely manner, or the adverse effects of utilizing inaccurate information have begun to appear. Therefore, healthcare institutional stakeholders should refer to these findings to prioritize strategies that guarantee the timeliness and accuracy of medical information quality, ensuring that proactive consumers can utilize information without such impediments.

Second, this study emphasizes the importance of accessibility and responsiveness in medical service quality. Our findings show that both accessibility and responsiveness significantly influence perceived usefulness and perceived ease of use. This indicates that consumers utilizing general hospitals perceive the service quality provided by these institutions as both useful and easy to process. These results are driven not only by the high quality of services offered by institutions but also by the evolving healthcare market. Historically, the medical market was supplier-centered, and consumers were passive recipients of limited information and services. However, as dissatisfaction with long waiting times, lack of communication with medical staff, and outdated facilities grew, consumers began seeking institutions that provide superior service quality. This reflects a paradigm shift from a supplier-centered to a consumer-centered medical market, where medical consumers have transitioned from passive subjects to proactive agents [44]. Consequently, there is an increasing strategic imperative to improve customer-oriented medical service quality for the survival of healthcare institutions [45]. Stakeholders must reflect these voices of consumers demanding high-quality care and focus on providing accessible and responsive services, particularly in the context of general hospitals.

Third, this study underscores that satisfaction is a critical element for both healthcare institutions and medical consumers. From the perspective of healthcare providers, satisfaction is a factor that enables the maintenance of continuous relationships with consumers and serves as an indicator for evaluating the quality of care. Since retaining existing patients is significantly more profitable for hospital management than recruiting new ones, satisfaction remains a paramount factor [20]. For medical consumers, satisfaction is equally vital as it is directly linked to health and life. Recently, with the development of information technology, consumers have begun to actively share their medical service experiences and provide feedback on their satisfaction levels to ensure they receive high-quality care [46,47]. Therefore, healthcare administrators must prioritize the enhancement of patient satisfaction by referring to both prior research and the empirical findings of this study.

### 5.3. Limitations and Future Research

Despite its significant academic and practical contributions, this study has several limitations that suggest directions for future research:

First, although this study analyzed consumers with experience in general hospitals, it did not distinguish between outpatient and inpatient services. Given that the nature of medical information and service quality perceptions may vary significantly depending on the type of care received, future studies should conduct detailed stratified analyses focusing specifically on the differing experiences of outpatients and inpatients.

Second, the scope of this research was limited to general hospitals. To enhance the generalizability of the findings, future research should encompass a broader range of healthcare settings, including primary clinics and public health centers, through comparative analyses that account for the unique characteristics of different types of medical institutions.

Third, this study did not categorize participants by their specific purpose of visit. Future research would benefit from investigating how service quality dimensions and technology acceptance vary according to the reason for visiting, such as treatment for illness, health promotion, or vaccination (e.g., COVID-19 or influenza).

Fourth, the study did not fully account for socio-demographic diversity, particularly in terms of occupation. In our sample (*N* = 376), office workers accounted for more than half of the participants (*n* = 199, 52.9%), which may introduce bias into the results. Subsequent studies should aim for a more balanced representation across various occupational groups to ensure a more comprehensive understanding of medical consumer behavior.

Fifth, the study relies on a cross-sectional and self-reported survey design, and Harman’s single-factor test yielded a variance of 61.71%, which is a recognized warning sign for common method bias (CMB). Although our confirmatory factor analysis and discriminant validity tests confirmed sufficient construct distinctiveness, the self-reported nature of the data means that common method variance could potentially overestimate some of the causal pathways. Future longitudinal research incorporating objective administrative data or applying procedural separation techniques during data collection is highly recommended to validate these relationships and overcome the inherent boundaries of single-source cross-sectional data.

## 6. Conclusions

In conclusion, this study validates the critical shift toward “proactive consumers” in the evolving healthcare landscape. By integrating medical information quality and service quality into the Technology Acceptance Model (TAM), we have demonstrated that enhancing accuracy, responsiveness, and accessibility is essential for fostering patient satisfaction and long-term revisit intentions. As the healthcare market continues to become more consumer-centered, hospital administrators must prioritize these quality dimensions to maintain institutional trust and ensure sustainable management in the digital age.

## Figures and Tables

**Table 1:** Operational Definition of Variables.

Variable	Operational Definition	References
Medical Information Quality—Timeliness	The degree to which medical information obtained through information seeking is perceived as up-to-date and timely for use.	[8,9]
Medical Information Quality—Accuracy	The degree to which medical information obtained through information seeking is perceived as accurate and free of errors.	
Medical Service Quality—Accessibility	The degree to which a medical institution is perceived as being in a convenient location.	[14,15]
Medical Service Quality—Responsiveness	The degree to which medical institution staff provide prompt answers.	
Perceived Usefulness	The degree to which medical information and services are perceived as beneficial and helpful.	[16,17]
Perceived Ease of Use	The degree to which medical information and services are perceived as being easy to use and capable of being used proficiently without difficulty.	
Satisfaction	The degree of satisfaction with the corresponding medical institution.	[20]
Revisit Intention	The degree to which a person intends to continue using the corresponding medical institution.	[24]

**Table 2:** Demographic Characteristics.

Characteristic	Category	Frequency (*n*)	Percentage (%)
Gender	Male	206	54.8
	Female	170	45.2
Age	20s	73	19.4
	30s	156	41.5
	40s	65	17.3
	50s	48	12.8
	60s and above	34	9.0
Occupation	Office worker	199	52.9
	Self-employed	41	10.9
	Professional	60	16.0
	Public official	62	16.5
	Others	14	3.7
Monthly Income (KRW)	Less than 2,000,000	11	2.9
	2,000,000–2,999,999	103	27.4
	3,000,000–3,999,999	155	41.2
	4,000,000–4,999,999	54	14.4
	5,000,000 and above	53	14.1
Total		376	100

**Table 3:** Confirmatory Factor Analysis Results.

Construct	Factor Loadings	Cronbach’s α	AVE	CR	VIF
Timeliness	0.928	0.940	0.80	0.94	3.939
	0.908				
	0.870				
	0.872				
Accuracy	0.921	0.959	0.86	0.96	4.565
	0.918				
	0.910				
	0.950				
Accessibility	0.862	0.935	0.78	0.93	2.887
	0.944				
	0.901				
	0.820				
Responsiveness	0.932	0.960	0.86	0.96	3.204
	0.932				
	0.929				
	0.910				
Perceived Usefulness	0.927	0.963	0.87	0.96	3.951
	0.935				
	0.927				
	0.933				
Perceived Ease of Use	0.920	0.956	0.84	0.96	4.107
	0.932				
	0.928				
	0.896				
Satisfaction	0.934	0.959	0.83	0.95	3.727
	0.907				
	0.932				
	0.859				
Revisit Intention	0.939	0.943	0.81	0.95	
	0.947				
	0.902				
	0.814				

Note: Revisit Intention is the final endogenous variable in the structural model and does not act as a predictor; hence VIF is not applicable. χ^2^/df = 2.883, GFI = 0.887, TLI = 0.930, CFI = 0.938, SRMR = 0.027, RMSEA = 0.074; AVE = average variance extracted; CR = composite reliability; VIF = variance inflation factor.

**Table 4:** Discriminant Validity Analysis Results.

	Timeliness	Accuracy	Accessibility	Responsiveness	Perceived Usefulness	Perceived Ease of Use	Satisfaction	Revisit Intention
Timeliness	(0.895)							
Accuracy	0.844 **	(0.925)						
Accessibility	0.726 **	0.768 **	(0.883)					
Responsiveness	0.555 **	0.563 **	0.598 **	(0.926)				
Perceived Usefulness	0.687 **	0.752 **	0.742 **	0.702 **	(0.931)			
Perceived Ease of Use	0.648 **	0.632 **	0.656 **	0.849 **	0.751 **	(0.919)		
Satisfaction	0.695 **	0.741 **	0.707 **	0.697 **	0.836 **	0.741 **	(0.909)	
Revisit Intention	0.611 **	0.621 **	0.596 **	0.589 **	0.650 **	0.644 **	0.723 **	(0.902)

Note: Diagonal values (in parentheses) are the square roots of the AVE. ** *p* < 0.01.

**Table 5:** Hypothesis Testing Results.

Path			Estimate	S.E.	C.R.	*p*	Decision
H1a: Timeliness	→	Perceived Usefulness	0.044	0.05	0.871	0.384	Not Supported
H1b: Accuracy	→	Perceived Usefulness	0.338	0.056	5.983	<0.001	Supported
H2a: Timeliness	→	Perceived Ease of Use	0.206	0.049	4.211	<0.001	Supported
H2b: Accuracy	→	Perceived Ease of Use	0.017	0.055	0.312	0.755	Not Supported
H3a: Accessibility	→	Perceived Usefulness	0.261	0.047	5.597	<0.001	Supported
H3b: Responsiveness	→	Perceived Usefulness	0.258	0.035	7.338	<0.001	Supported
H4a: Accessibility	→	Perceived Ease of Use	0.135	0.046	2.944	0.003	Supported
H4b: Responsiveness	→	Perceived Ease of Use	0.655	0.034	19.021	<0.001	Supported
H5a: Perceived Usefulness	→	Satisfaction	0.652	0.042	15.684	<0.001	Supported
H5b: Perceived Ease of Use	→	Satisfaction	0.229	0.038	6.089	<0.001	Supported
H6a: Perceived Usefulness	→	Revisit Intention	0.01	0.073	0.143	0.886	Not Supported
H6b: Perceived Ease of Use	→	Revisit Intention	0.261	0.053	4.885	<0.001	Supported
H7: Satisfaction	→	Revisit Intention	0.527	0.07	7.506	<0.001	Supported

Note: S.E. = standard error; C.R. = critical ratio.

**Table 6:** Bootstrapping Intermediary and Indirect Effects.

Interventions & Paths	Indirect Effect	S.E.	95% CI (Lower)	95% CI (Upper)	*p*-Value	Result
Perceived Usefulness → Satisfaction → Revisit Intention	0.342	0.059	0.232	0.464	<0.001	Significant (Full Mediation)
Perceived Ease of Use → Satisfaction → Revisit Intention	0.12	0.026	0.073	0.174	<0.001	Significant (Partial Mediation)

Note: S.E. = standard error.

## Data Availability

The data presented in this study are available on request from the corresponding author due to ethical restrictions regarding participant anonymity and privacy. The raw data are not publicly available to ensure compliance with institutional ethics guidelines.
